# Two-Dimensional Self-Assembly Driven by Intermolecular Hydrogen Bonding in Benzodi-7-azaindole Molecules on Au(111)

**DOI:** 10.1021/acs.jpcc.3c01640

**Published:** 2023-06-08

**Authors:** José Abad, José I. Martínez, Paula Gómez, Miriam Más-Montoya, Luis Rodríguez, Albano Cossaro, Alberto Verdini, Luca Floreano, José A. Martín-Gago, David Curiel, Javier Méndez

**Affiliations:** † Applied Physics Department, Technical University of Cartagena, c/ Dr. Fleming s/n, 30202 Cartagena, Spain; ‡ Department of Low Dimensional Systems, Institute of Materials Science of Madrid (ICMM-CSIC), c/ Sor Juana Inés de la Cruz 3, 28049 Madrid, Spain; § Department of Organic Chemistry, Faculty of Chemistry, University of Murcia, 30100 Murcia, Spain; ∥ CNR-IOM, Laboratorio TASC, 34149 Trieste, Italy; ⊥ Department of Chemical and Pharmaceutical Sciences, University of Trieste, Trieste I-34149, Italy

## Abstract

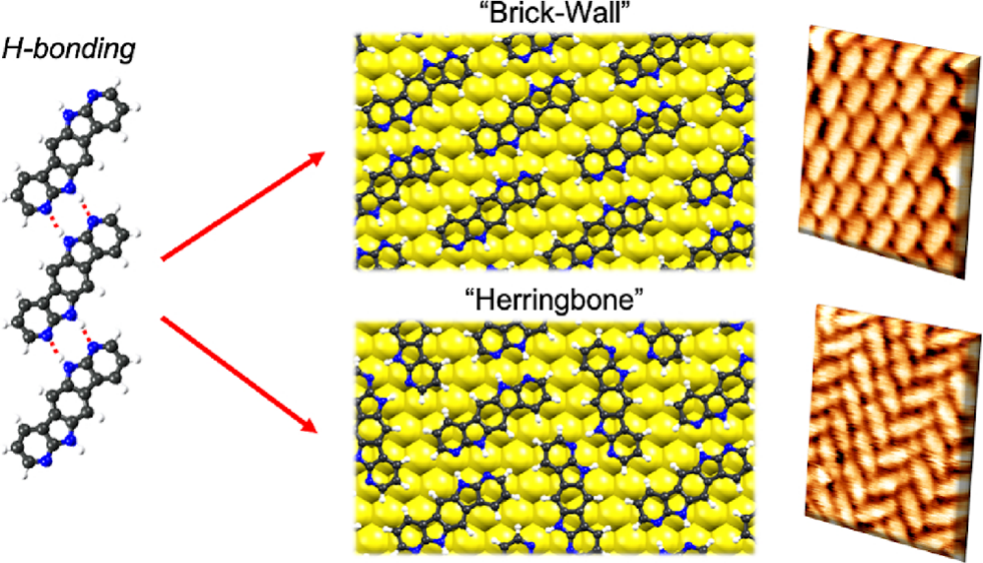

The control of molecular structures at the nanoscale plays a critical role in the development of materials and applications. The adsorption of a polyheteroaromatic molecule with hydrogen bond donor and acceptor sites integrated in the conjugated structure itself, namely, benzodi-7-azaindole (**BDAI**), has been studied on Au(111). Intermolecular hydrogen bonding determines the formation of highly organized linear structures where surface chirality, resulting from the 2D confinement of the centrosymmetric molecules, is observed. Moreover, the structural features of the **BDAI** molecule lead to the formation of two differentiated arrangements with extended brick-wall and herringbone packing. A comprehensive experimental study that combines scanning tunneling microscopy, high-resolution X-ray photoelectron spectroscopy, near-edge X-ray absorption fine structure spectroscopy, and density functional theory theoretical calculations has been performed to fully characterize the 2D hydrogen-bonded domains and the on-surface thermal stability of the physisorbed material.

## 1 Introduction

Molecular organization has a critical effect on the physical, chemical, and biological properties of organic materials with applications in areas as diverse as electronics, photonics, catalysis, or biomedicine.^1−5^ In fact, the control of matter and processes at the nanoscale represents the essence of nanotechnology.^6^ Nevertheless, concerning the arrangement of organic materials, since the structure of molecular solids is governed by very weak noncovalent interactions, it is currently very difficult to set a reliable structure–property correlation that can predict the organization in the solid state from the structure of the molecule.^7−9^ With the aim of gaining a better understanding of the subtle energy balance that leads to a certain molecular organization, great efforts have been made in the design of molecules that can be used as models to be studied in the areas of crystal engineering.^10,11^ These circumstances that have been extensively addressed in 3D structures are equally applicable and have lately received much attention concerning the 2D molecular ordering on surfaces.^12−14^ In this case, apart from the molecule–molecule interactions, the confinement into a two-dimensional environment implies that the molecule–substrate interactions, controlling the organic material physisorption, become an indispensable aspect to be considered in the structural characterization.^15^ One of the approaches that can contribute to a better control of the molecular organization is the supramolecular self-assembly.^16−18^ In this regard, hydrogen bonding becomes a useful tool due to the higher energy and directionality of hydrogen bonds when compared to other noncovalent interactions.^19^ Thus, the strategic location of hydrogen bonding sites in a molecule can be used to control the thermodynamics of intermolecular interactions, partially influencing the growth of molecular nanostructures through a bottom-up approach.^20−22^

Focusing our attention on conjugated molecules, which have gained much relevance due to their use as semiconductors in different electronic applications (organic field-effect transistors, organic light-emitting devices, or organic and hybrid solar cells), their charge transport properties are determined by their intermolecular interactions and their disposition at the substrate interface.^23−25^ Therefore, the characterization of the molecular arrangement on surfaces provides essential information for the interpretation of material properties and for the development of novel materials.^26−28^ Scanning tunneling microscopy (STM) becomes a particularly valuable technique for the study of self-assembled nanostructures on surfaces, given the degree of detail that can be reached with molecular or atomic resolution.^29,30^ In this regard, it is worth highlighting the results reported for hydrogen-bonded conjugated systems with application in the area of organic electronics. Quinacridone has been comprehensively studied on different surfaces such as highly oriented pyrolytic graphite (HOPG), Ag(111), Ag(100), and Cu(111).^31−33^ Although different degrees of strength in the adsorption of quinacridone have been observed depending on the molecule–substrate interactions, in all cases, homochiral linear structures were observed for each of the expected surface enantiomers. This arrangement results from the complementary hydrogen bonds set between the carbonyl and the NH groups present in the structure of quinacridone. Similar results were reported for the surface self-assembly of indigo on Cu(111), where enantiopure one-dimensional chains were observed.^34^ Diketopyrrolopyrrole (DPP) is another building block frequently used in the synthesis of organic semiconductors. Its structure also contains two carbonyl groups and two NHs representing the same hydrogen bonding motif as quinacridone and indigo. The short conjugation length of DPP can be easily enlarged by attaching aromatic substituents. These derivatives form hydrogen-bonded linear structures intercalated with solvent molecules when deposited from long alkanoic acid solutions on HOPG.^35,36^ The *N*-heteroacene dihydrotetraazapentacene constitutes another conjugated molecule whose ability to self-assemble on Au(111), Cu(110), and c-plane sapphire surfaces has been studied.^37−39^ In this case, the N–H···N interactions between tetrahydropyrazine and pyrazine-like nitrogens induce the formation of well-ordered molecular rows that grow in homochiral domains.

**Figure 1:**
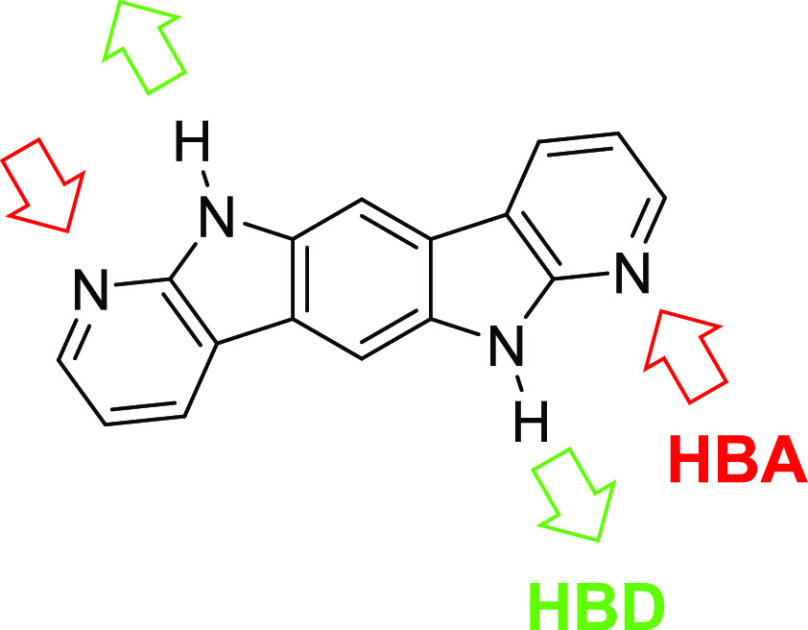
Structure of 6,12-dihydrobenzo[1,2-*b*:4,5-*b’*]di(7-azaindole), **BDAI**. HBD: hydrogen bond donor site; HBA: hydrogen bond acceptor site.

Within this context, we have recently reported a supramolecular approach based on the surface self-assembly of a conjugated tripodal system that formed expanded enantiopure domains.^40^ The self-resolution resulting from the formation of energetically favored hydrogen-bonded hexamers led to a two-dimensional framework. The reciprocal hydrogen bonding set through strategically located 7-azaindole units has revealed its suitability as a building block for controlling the molecular disposition of conjugated systems as we have demonstrated in different electronic applications.^41,42^ Accordingly, herein we report the integration of this building block into a structurally related molecule, namely, benzodi-7-azaindole (**BDAI**), resulting from the condensation of two 7-azaindole units to a central benzene core (Figure 1). This system reinforces the hydrogen bonding by using a stronger base as an acceptor site (N_pyridine_···H-N_pyrrole_) and differs from most of the previously reported molecules based on C=O···HN interactions. Moreover, the fused 7-azaindole building block leads to fully conjugated systems that contrast the cross-conjugation commonly observed in most of the abovementioned compounds.

The surface chirality emerging from the adsorption of this molecule with a centrosymmetric structure can produce two surface enantiomers when deposited on Au(111). Ideally, the molecules can form extended unidirectional hydrogen-bonded structures. Besides, the lateral packing of these structures leads to expanded domains with either brick-wall or herringbone arrangement depending on the homochiral or racemic composition, respectively. In this work, we use several surface-science experimental techniques, including STM, high resolution X-ray photoelectron spectroscopy (HR-XPS), and near-edge X-ray absorption fine structure spectroscopy (NEXAFS), combined with density functional theory (DFT) calculations, to get detailed characterization of the geometry and bonding of self-assembled 2D monolayers of **BDAI** molecules on Au(111), in ultrahigh vacuum (UHV), as well as their thermal stability. The chosen substrate Au(111) provides a weak substrate–molecule interaction and allows the observation of molecular structures close to a free-standing situation.

## 2 Experimental Section

The synthesis of 6,12-dihydrobenzo[1,2-*b*:4,5-*b’*]di(7-azaindole), **BDAI**, has been reported elsewhere.^43^ The preparation and characterization of physisorbed layers have been performed in two different UHV systems: a home-laboratory with STM, low energy electron diffraction (LEED), quadrupole mass spectrometry (QMS), and Auger electron spectroscopy (AES) techniques, and a synchrotron laboratory for HR-XPS and NEXAFS.

### 2.1 Scanning Tunneling Microscopy (STM)

A gold surface was prepared by sputtering and annealing cycles of a Au(111) single crystal. Cleanliness was checked by AES and LEED using a for-grid SPECTALEED from Omicron. Pre-purified **BDAI** molecules were evaporated in UHV from a tantalum crucible, and the evaporation rates were measured using a quartz balance. Typically, low evaporation rates about 0.1 Å/min were used while monitoring the process via a quadrupolar mass spectrometer (QMS). The sample prepared was transferred to the STM chamber. The images were obtained using an Omicron VT-STM operated with Nanotec’s WSxM^44^ software and electronics. STM images were acquired at several temperatures in a range between room temperature and 50 K without substantial differences. STM tips used were made of electrochemical etched W wire, pre-cleaned by annealing in vacuum and self-sputtering processes.^45^

### 2.2 High-Resolution X-ray Photoelectron Spectroscopy (HR-XPS) and Near-Edge X-ray Absorption Fine Structure (NEXAFS)

The HR-XPS and NEXAFS experiments were performed at the ALOISA beamline, at Elettra synchrotron.^46^ For the HR-XPS and NEXAFS measurements, the deposition rate was checked during deposition with a quartz crystal microbalance. The typical deposition rate was 0.1 Å/min for monolayer films and 0.4 Å/min for multilayer ones. The monolayer coverage (ML) was found to correspond to an effective thickness of 2.5 Å, as determined from the residual XPS intensity after thermal desorption of a multilayer. The C and N K-edge spectra were taken in electron yield mode using a channeltron detector,^46^ and they were further analyzed following the procedure described in the literature.^47^ The orientation of the surface with respect to the linear polarization of the synchrotron beam was changed by rotating the sample around the beam axis while keeping a constant grazing angle of 6°. This scattering geometry allows the change from linear p-polarization (light polarization perpendicular to the sample surface) to linear s-polarization (light polarization parallel to the sample surface) without any variation of the illuminated area on the sample. The photoemission spectra were taken with the X-ray beam impinging on the sample at a grazing incidence angle (4°) at two photon energies, namely, 400 and 515 eV, to measure the XPS core levels Au 4f, C 1s, and N 1s. The binding energy (BE) scale was calibrated with respect to the Au 4f_7/2_ substrate peak at 84.00 eV. The background due to inelastically scattered photoelectrons has been subtracted from raw data by a Shirley background routine, the convolution of a Gaussian and a Lorentzian function has been used to fit the photoemission peaks, and more details about the fitting procedure are given in the SI. During the synchrotron measurements, to minimize the beam-induced damage, the sample was continuously displaced.

The Au(111) surface was prepared by standard sputtering and annealing procedures. The cleanliness of the surface before deposition was checked by AES and HR-XPS.

### 2.3 Computational Details

On the basis of the experimental LEED and STM evidences, the structure, electronic properties, and theoretical STM imaging of the two different **BDAI** molecular phases on Au(111) observed have been theoretically investigated by density functional theory (DFT) by the use of an adequate combination of the plane-wave and localized basis set DFT-based atomistic simulation packages QUANTUM ESPRESSO^48^ and FIREBALL^49^ (see further details in the SI). To make a direct comparison with the XPS experimental results for the different phases, calculation of N 1s core level binding energy shifts (CLS) has been performed with the plane-wave code QUANTUM ESPRESSO^48^ within the final state approximation^50^ (see the Supporting Information).

## 3 Results and Discussion

### 3.1 STM and Computational Results

**Figure 2:**
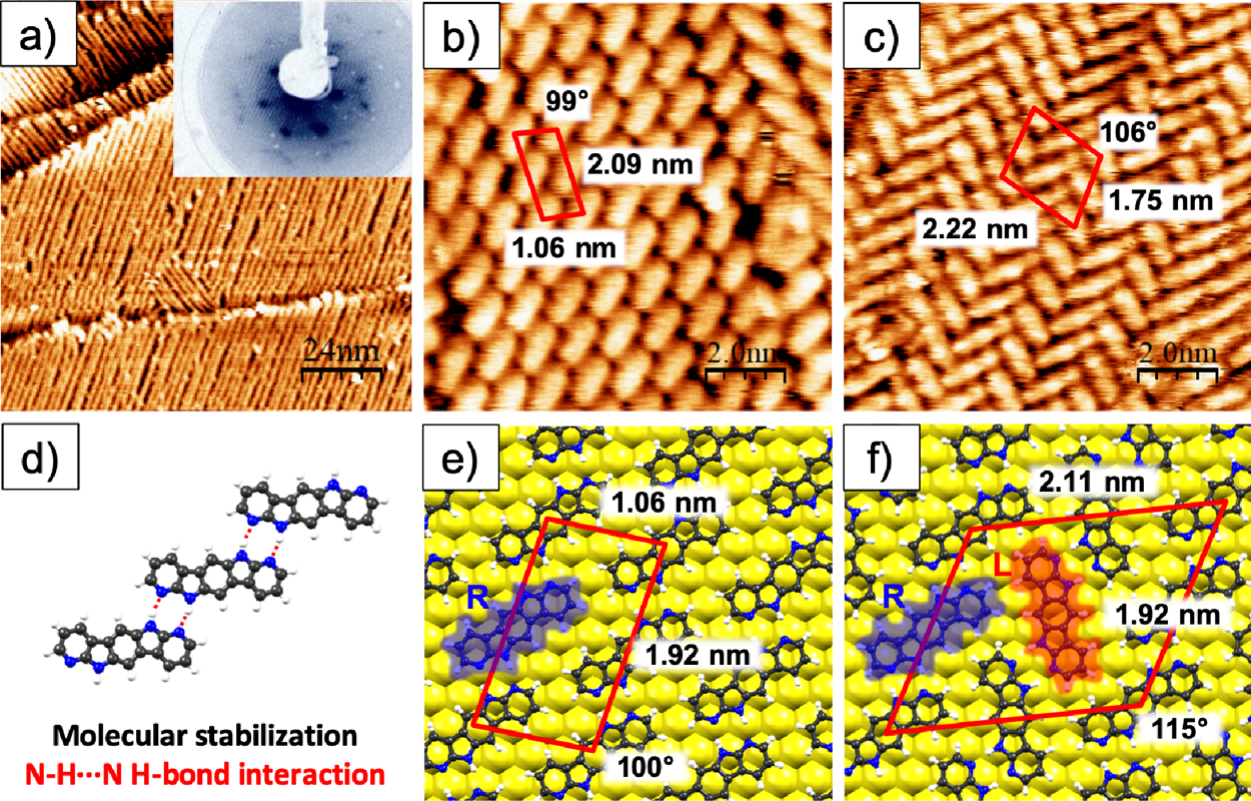
(a) STM image of the quasi-one-dimensional rows (120 × 120) nm^2^. The inset shows the LEED pattern at *E* = 21.3 eV. (b) STM images (10 × 10) nm^2^ of brick-wall packing and (c) herringbone packing of the **BDAI** molecule at a constant-current regime (*I*_tunnel_ = 50 nA and *V*_bias_ = 100 mV). (d) Formation of the order unit, a chain of homochiral molecules, through N–H···N bonds. (e) Homochiral and (f) racemic pictorial top views of both DFT fully structurally optimized molecular phases, with R and L enantiomers marked in blue and red, respectively.

The first evidence of the formation of ordered assemblies of **BDAI** on gold is obtained by LEED. Extra spots, in registry with the Au(111) LEED reflections, appear in the pattern after submonolayer evaporation of **BDAI**, as it is shown in the inset of Figure 2a. STM images, recorded at this particular molecular coverage, show ordered structures (Figure 2a) arranged in quasi-one-dimensional molecular rows. Centrosymmetric **BDAI** molecules become chiral when deposited flat on a surface. Given the restrictions imposed by the impossibility of surface flipping, depending on the face of the molecule that gets physisorbed, two possible surface enantiomers originate. The observed rows are ascribed to one of these enantiomers. The reciprocal N–H···N interactions between neighboring molecules induce a chiral selectivity allowing only the precise enantiomer to form a whole row of molecules with the same chirality. These rows became the structural unit of the **BDAI** self-assembling (Figure 2d) and grow preferentially following the gold reconstruction lines (Figure S1 in the Supporting Information), namely, along the [112̅] directions. An increase in coverage produces a further packing of the homochiral rows, with hydrogen bonding between adjacent molecules, extending laterally the assembly. The formation of enantiopure domains has been typically observed for other centrosymmetric molecules whose hydrogen bonding on the surface has been characterized.^33,38^ Interestingly, the particularities of the **BDAI** structure, with a pseudolinear skeleton and the hydrogen bonding sites located at both ends of the polyheteroaromatic system, lead to a self-assembled structure with a noticeable intermolecular shift within the supramolecular rows. As a consequence, these rows can adopt two different molecular arrangements distinguishable in the STM images: the brick-wall packing (Figure 2b) and the herringbone packing (Figure 2c). The brick-wall domains correspond to hydrogen-bonded rows constituted by molecules with the same surface chirality (Figure 2b,e) that we assigned to the R enantiomer (marked in blue in Figure 2e). Figure 2e exemplifies the domain that we assigned to the R enantiomer (highlighted in blue). Differently, the herringbone domains are formed by homochiral hydrogen-bonded rows that are alternated with rows of the opposite surface chirality, producing an extended racemic coverage (Figure 2c,f), with R and L enantiomers (rotated rightward or leftward, respectively, with respect to the direction of the gold reconstruction line).

The unit cell obtained for the brick-wall arrangement is (1.06 × 2.09) nm^2^ with an angle of 99°, while in the herringbone arrangement, the unit cell is (1.75 × 2.22) nm^2^ with an angle of 106°. In the latter packing, some rows of molecules are narrower, probably due to electronic effects. In both domains, intermolecular stabilization of the adlayers is mainly driven by complementary donor–acceptor N–H···N hydrogen bonds between adjacent molecules in the rows (with average N···N distances of 2.95 and 2.85 Å for the brick-wall packing and herringbone phases, respectively). Additionally, different edge-to-edge intermolecular interactions (with distances ranging between 2.2 and 2.6 Å) contribute to the lateral packing of homochiral or enantiomer rows producing densely packed domains in both phases. Figure 2e,f shows the DFT results corresponding to the full structural optimization of these molecular domains. Resulting interfaces reveal very close molecular arrangements as compared with the experimental STM images, fully consistent with the representative distances extracted from them. In both phases, the interaction between the molecular adlayer and the Au(111) substrate has an eminent van der Waals character, with no subtle chemistry underlying (computed molecule/substrate charge transfer < 0.05e^–^). The interaction with the substrate in this physisorption regime is intense enough to anchor the molecules to the surface but not sufficient to distort the gas-phase molecular morphology, which has its reflection in that the molecules of both phases are essentially flat on the surface (*vide infra* for NEXAFS discussion) at perpendicular distances of 3.19 and 3.32 Å and binding energies per molecule of −1.12 and −0.93 eV for the brick-wall and herringbone molecular arrangements, respectively. Binding energies have been obtained as the difference between the computed total energy of the whole interfacial systems and the sum of the total energies of the corresponding surfaces and molecular adlayers separately. This slight difference of 0.19 eV in the adsorption energies between both phases seems to arise from a more efficient packing of the brick-wall arrangement, which also manifests in the lower adsorption energy and a slightly lower adsorption molecular distance. The less robust packing of the herringbone adlayer favors a slightly lower decoupling degree from the substrate with respect to the brick-wall phase, thus decreasing the binding energy with the Au surface. Experimentally, in a rough analysis of the STM images, we also observe a preference for the brick-wall phase with around 67% of the covered areas, even though this homochiral structure requires segregation of the enantiomers.

**Figure 3:**
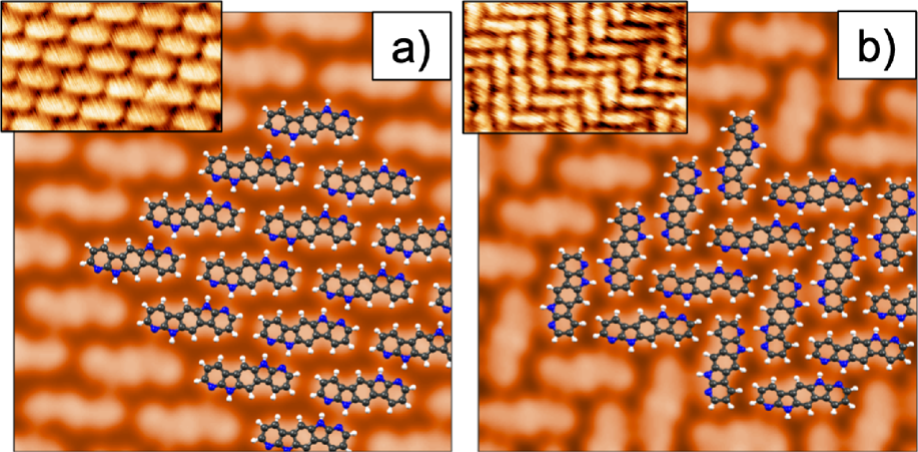
**BDAI** simulated STM images (a) of the brick-wall phase and (b) of the herringbone arrangement. The insets show the experimental STM images.

We have also simulated theoretical STM images based on the Keldish–Green formalism for the optimized interfacial models obtained under the constant-current regime as in the experiments. Excellent match between the experimental and simulated images has been obtained for both structures: brick-wall (Figure 3a) and herringbone (Figure 3b). The superposition of the structural adlayer models onto the simulated STM images allows the assignment of a molecule to each individual protrusion observed in the STM images. Intramolecular resolution is slightly higher in the simulated images than in the experimental ones since simulations are performed at 0 K without considering the effect of the environmental thermal bath, whereas STM images were recorded at 100 K.

### 3.2 HR-XPS Results

**Figure 4:**
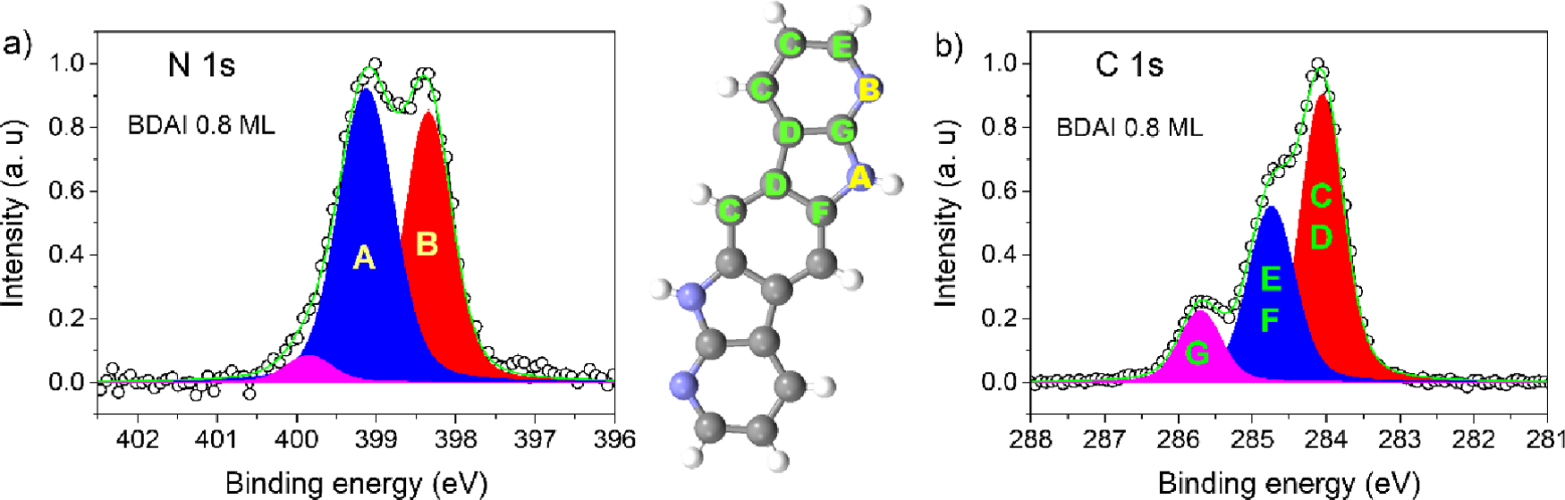
(a) N 1s and (b) C 1s XPS spectra for 0.8 ML of the **BDAI** molecule. Carbon and nitrogen atoms have been labeled according to their bond connectivity for the interpretation of XPS spectra.

The spectroscopic characterization of almost one physisorbed monolayer (0.8 ML or 2.0 Å) was carried out by HR-XPS probing both C 1s and N 1s levels (Figure 4). The N 1s core level for 0.8 ML of **BDAI** fitted with three components at binding energies of 398.35, 399.13, and 399.85 eV (Figure 4a); details of the fit are found in Table S1 of the Supporting Information. The areas of the more intense peaks are 43 and 53%, respectively, showing reasonable agreement, within the experimental error, with the presence of two non-equivalent N atoms in the molecule. The difference in energy between the main features is 0.78 eV, which matches the experimental value of 0.8 eV, found in the literature for the 2,3-dihydro-7-azaindole molecule in gas phase.^51^ The peak at 398.35 eV corresponds to N-pyridine as it has been previously reported in the literature at 398.00 eV,^52^ while the second peak is ascribed to the pyrrolic NH whose binding energy has been reported at about 400 eV.^52−54^ The decrease in the binding energy of this component with respect to the reported value, about 0.87 eV, is due to the formation of the intermolecular hydrogen bond. This interaction affects the polarization of the covalent N–H bond, producing a lower binding energy shift on the N 1s level with respect to the isolated molecule. For the same reason, the nitrogen atom in the pyridine ring experiences a shift to higher binding energies of 0.35 eV. Shifts in the binding energy of N acceptor atoms due to the formation of new hydrogen bonds have been previously reported in the literature.^55,56^

The small component located at 399.85 eV can be attributed to (−NH) that does not form H bonds or hydrogenated pyridine.^53^

Concerning the C 1s core level for the 0.8 ML on Au(111), the deconvolution of the spectra fitted well with three components located at 284.06, 284.74, and 285.70 eV with 52, 35, and 13% of relative weight, respectively (Figure 4b). The molecule presents eight nonequivalent carbon atoms, and, although it is not possible to solve the contribution of each C atom to the spectra, they can be grouped according to their interatomic connectivity. In agreement with these criteria, the C atoms bonded to C or H (type C and D) are ascribed to the peak with a lower binding energy (theoretically 63% of the total area), the C atoms bonded to one N atom (type E and F) are attributed to the second component (theoretically 25% of the total area), and finally, the C atoms bonded to two more electronegative N atoms (type G) correspond to the higher binding energy component (theoretically 13% of the total area). The areas of each component are in agreement with the different kinds of C atoms present in the molecule.

**Figure 5:**
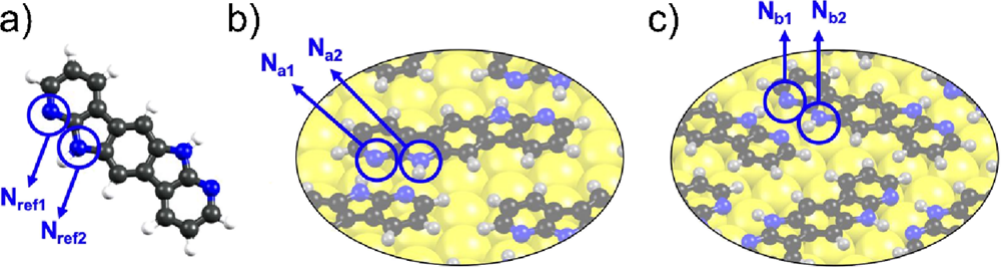
Pictorial view of the DFT-optimized systems considered for the calculations of the N 1s CLS. (a) Gas-phase **BDAI**, where the N atoms are not involved in any intermolecular bond and are taken as reference (N_ref1_ and N_ref2_). Trial systems: (b) brick-wall interfacial phase and (c) herringbone interfacial phase. N atoms of interest are indicated (N_a1_, N_a2_, N_b1_, and N_b2_) in (a), (b), and (c).

Theoretical DFT-based calculations of N 1s CLS have been carried out to rationalize the origin of the N 1s core level differences observed in the XPS experiments (Figure 5). The binding energy difference between N atoms in the pyridine and pyrrole rings has been computed for the two optimized **BDAI**/Au brick-wall (Figure 5b) and herringbone (Figure 5c) interfacial phases.

These calculations led to values of −0.42 and +0.21 eV for CLS_final_(N_a1_) and CLS_final_(N_a2_), behaving, as expected, as donor and acceptor sites, respectively. These values lead to a difference [CLS_final_(N_a2_) – CLS_final_(N_a1_)] of +0.63 eV with the pyrrolic N atom showing higher binding energy than the pyridinic one for the brick-wall domain. As far as the calculations for the herringbone domain are concerned, values of −0.39 and +0.32 eV were obtained for CLS_final_(N_b1_) and CLS_final_(N_b2_), behaving again as donor and acceptor sites, respectively. These values lead to a difference [CLS_final_(N_b2_) – CLS_final_(N_b1_)] of +0.71 eV, with the pyrrolic N having a higher binding energy. The computed values of +0.63 and +0.71 eV agree with the experimental XPS result of +0.78 eV for the difference between N atoms in the pyrrole and pyridine rings.

### 3.3 NEXAFS Results

**Figure 6:**
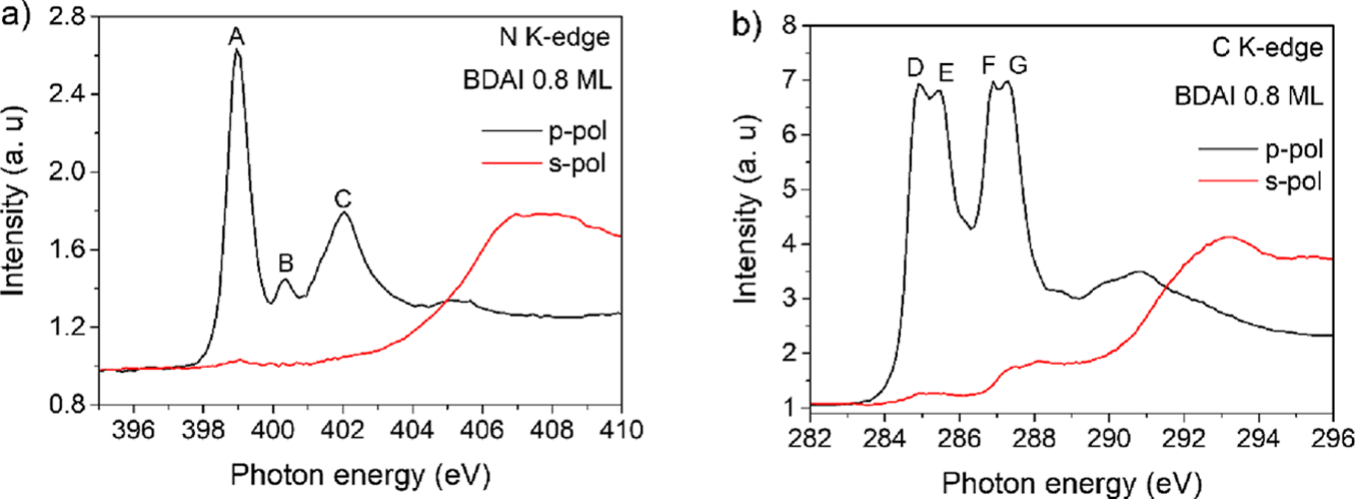
NEXAFS spectra of 0.8 ML **BDAI** deposited on Au(111), collected at the (a) N K-edge and (b) C K-edge, with the electric field of the incident photons perpendicular (p-polarization) to the surface (black line) and with the electric field of the incident photons parallel (s-polarization) to the surface (red line).

We performed linearly polarized C and N K-edge NEXAFS measurements to elucidate the chemical and geometrical properties of the molecules on Au(111). The p- and s-polarized NEXAFS spectra of the 0.8 ML **BDAI** film are shown in Figure 6. The p-polarized N K-edge spectrum (Figure 6a) presents the most intense peak at 398.95 eV (peak A), which can be associated with a N 1s → π* transition, localized solely on the pyridinic nitrogen (C=N-C), in full agreement with gas-phase measurements on pyridine^57^ and azaindole.^58^ From comparison with the literature on pyrrole,^59^ azaindole,^58^ and imidazole,^60^ we may associate the broad and asymmetric resonance peak at 402.05 eV (peak C) with the lowest energy contribution from the pyrrolic nitrogen atom, corresponding to a N 1s → π* (N–H) transition. The assignment of the small absorption feature at 400.36 eV (peak B) is less straightforward. We can exclude an interfacial origin (i.e., rehybridization with the substrate) because it is also observed in multilayers (grown at low temperatures) with similar weight (see Figure S5 of the Supporting Information). A relatively weaker shoulder of the main peak at 1.4 eV higher energy was reported in the case of pyridine.^57^ Former NEXAFS measurements on imidazole also reported a clear peak in between the two main C=N-C and N–H π* resonances, as due to the transition of N 1s electrons (C=N-C) to the 2π* orbital.^60^ We may speculate that the sharper appearance of resonance B in the **BDAI** spectra with respect to azaindole may be associated with the overall increase in the spatial spread of the molecular orbitals due to the conjugation of the benzene ring.

Polarized C and N K-edge NEXAFS measurements were performed to elucidate the chemical and geometrical properties of the molecules on Au(111). The p-polarized and s-polarized NEXAFS spectra of the 0.8 ML **BDAI** film are shown in Figure 6.

Regarding the p-polarized C K-edge spectrum (Figure 6b), the first component observed at 285.0 eV (peak D) is ascribed to both the benzene and pyridine rings of the molecule, and the second component at 285.5 eV (peak E) corresponds to the pyridine and pyrrole rings. The two components are almost equivalent in the **BDAI** molecule, and they correspond to the C 1s transitions into the LUMO. The components at 286.8 eV (peak F) and at 287.3 eV (peak G) correspond to the C 1s transitions into the LUMO+1 states. The LUMO+1 is quite similar in shape and intensity to the LUMO. Finally, the broad peak located at 290.8 eV is assigned to a σ* symmetry resonance.

The relatively sharp C and N K-edge peaks are evidence of the weak interaction between molecules and the substrate as it is expected for the Au(111) surface. When the polarization dependence of the NEXAFS spectra is analyzed, a strong dichroism is observed. The π* transitions show the maximum intensity when the electric field is almost normal to the surface, p-polarization, and their intensity decreases considerably when the electric field is parallel to the surface, s-polarization. The reverse behavior is observed for the σ* symmetry resonance. Therefore, we can determine the average orientation of the molecules relative to the surface through the refs (61, 62). The **BDAI** NEXAFS C K-edge shows that the average tilt angle of the molecules with respect the surface plane is 3.2° ± 1.5° for almost one monolayer. We also measured the NEXAFS for less than one monolayer (0.8 Å), and the average tilt of the molecules is 3.6° ± 1.7°. These low average tilt angles are indicative of molecules lying essentially flat on the substrate surface and show good agreement with the DFT calculations.

### 3.4 Thermal Stability

**Figure 7:**
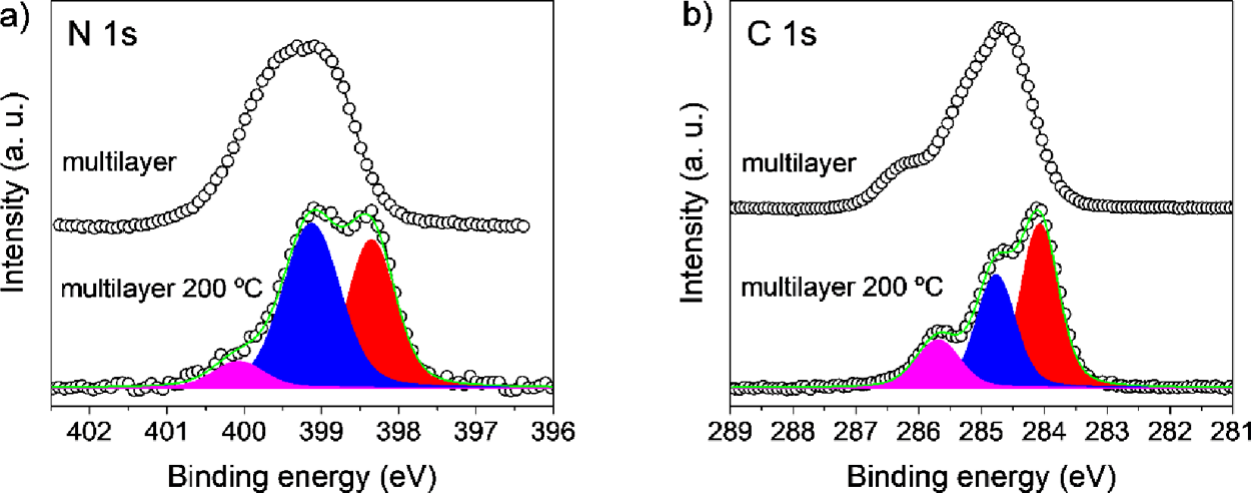
(a) N 1s and (b) C 1s XPS spectra for a multilayer of **BDAI** before and after annealing at 200 °C.

High thermal stability is a requirement that conjugated materials must meet for any optoelectronic application. Accordingly, the evolution of **BDAI** HR-XPS spectrum at different coverages, monolayer and multilayer, has been studied at different temperatures. Regarding the core level N 1s measurements for a **BDAI** multilayer, coverage of 11.7 Å, deposited at −80 °C, the spectrum shows one single broad component at 399.20 eV (Figure 7a). When this multilayer is annealed at 200 °C, the resulting profile resembles that of the monolayer, with three peaks observed at binding energies of 398.35, 399.13, and 400.07 eV, whose areas are 40, 52, and 8%, respectively. For the multilayer regime, the C 1s spectrum exhibits three components. However, these are wider than the monolayer sample, located at 284.51, 285.17, and 286.28 eV with 45, 45, and 10% of the total envelope, respectively. When the multilayer is annealed up to 200 °C (Figure 7b), a monolayer is formed and the C 1s core level matches to the shape of the previously presented spectrum for 0.8 ML (Figure 4b).

**Figure 8:**
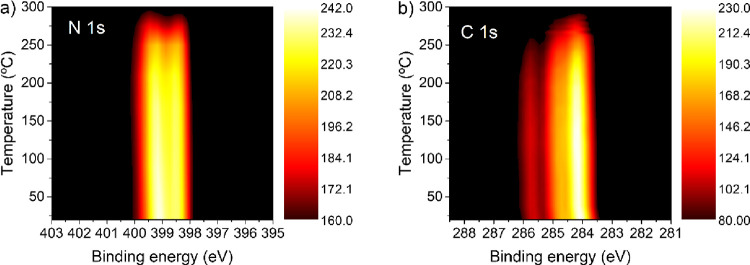
(a) N 1s and (b) C 1s 2D XPS spectra as a function of temperature for a monolayer of **BDAI**.

Finally, the thermal behavior of 1 ML has been analyzed following the evolution of the N 1s and C 1s core levels upon increasing the temperature in real time. Figure 8a shows a 2D image of the N 1s spectrum characterized by two peaks that correlate to the more intense components analyzed in detail in Figure 4a. When the sample is gradually annealed, the high binding energy component, ascribed to the pyrrolic N, shifts to higher energies with respect to the initial sample according to the following trend: 0.2 eV (90 °C), 0.3 eV (250 °C), 0.5 eV (275 °C), and 0.6 eV (300 °C). The molecules finally desorb at temperatures over 300 °C.

Concerning the C 1s spectrum for 1 ML (Figure 8b), it presents the three features analyzed above (Figure 4b). There are no significant changes in the lineshape and the binding energy of the spectra until 290 °C. At this temperature, the higher binding energy component disappears, and finally, at temperatures higher than 300 °C, the molecules are completely desorbed. These results show the high thermal stability of the **BDAI** hydrogen-bonded molecules in the physisorbed layer and the adequacy of this property to the stability requirements of electronic applications.

## 4 Conclusions

In summary, we have combined first-principles calculations with HR-XPS, NEXAFS, and STM experimental data to investigate the hydrogen bond-directed self-assembly of **BDAI** when adsorbed on Au(111). Two types of 2D chiral domains, namely, brick-wall and herringbone, stabilized by complementary N–H···N hydrogen bonding have been observed for **BDAI** molecules. NEXAFS and DFT experiments have revealed that the molecules lie essentially flat on the surface. The spectroscopic characterization of the flat monolayer by HR-XPS has confirmed the composition of the 2D self-assembled supramolecular structure and more importantly has revealed the effect of hydrogen bonding on the absolute binding energies shifts measured for N atoms in the pyridine and pyrrole rings. Finally, the thermal desorption studied at different coverages has proved the high stability of hydrogen-bonded monolayers formed by **BDAI**. These results reinforce the utility of hydrogen bonding as a tool for controlling the molecular organization at the nanoscale and improving the robustness of conjugated materials.

## Supporting Information Available

Surface characterization, STM and LEED, XPS fitting procedure, NEXAFS spectra of the multilayer, theoretical methods, and some representative structural models (PDF)
