# Supplementary material for: Two-Dimensional Self-Assembly Driven by Intermolecular Hydrogen Bonding in Benzodi-7-azaindole Molecules on Au(111)
Source: J Phys Chem C Nanomater Interfaces. 2023 Jun 8;127(24):11591–9. doi: 10.1021/acs.jpcc.3c01640 (PMC10291637; doi:10.1021/acs.jpcc.3c01640)
Supplement: Supplementary file 1 — jp3c01640_si_001.pdf [file jp3c01640_si_001.pdf]

2D Self-Assembly Driven by Intermolecular Hydrogen-Bonding in Benzodi-7-Azaindole Molecules on Au(111)

José Abad[1], José I. Martínez[2], Paula Gómez[3], Miriam Más-Montoya[3], Luis Rodríguez[2], Albano Cossaro[4,5], Alberto Verdini[4], Luca Floreano[4], José A. Martín-Gago[2], David Curiel*[,3] and Javier Méndez*[,2]

[1]Applied Physics Department, Technical University of Cartagena, c/ Dr. Fleming s/n, 30202 Cartagena (Spain)

[2]Department of Low Dimensional Systems, Institute of Material Science of Madrid (ICMM-CSIC), c/ Sor Juana Inés de la Cruz 3, 28049 Madrid (Spain)

[3]Department of Organic Chemistry, Faculty of Chemistry, University of Murcia, 30100-Murcia (Spain)

[4]C.N.R.-INFM, Laboratorio Nazionale TASC, Trieste (Italy)

[5]Department of Chemical and Pharmaceutical Sciences, University of Trieste, Trieste (Italy)

* davidcc@um.es j.mendez@csic.es

Supporting Information

Surface characterization. STM and LEED……………………………………….. S2

XPS fitting procedure…………………………………………………….….... S5

NEXAFS spectra of the multilayer……………………………………………..S6

Theoretical methods……………………………………………………………S6

Some representative structural models………..…..……………………………...S8

References………………………………………………………………………S9

Surface characterization

STM images were taken at different stages of the molecular growth. The following images represent the gold surface after submonolayer growth of **BDAI**.

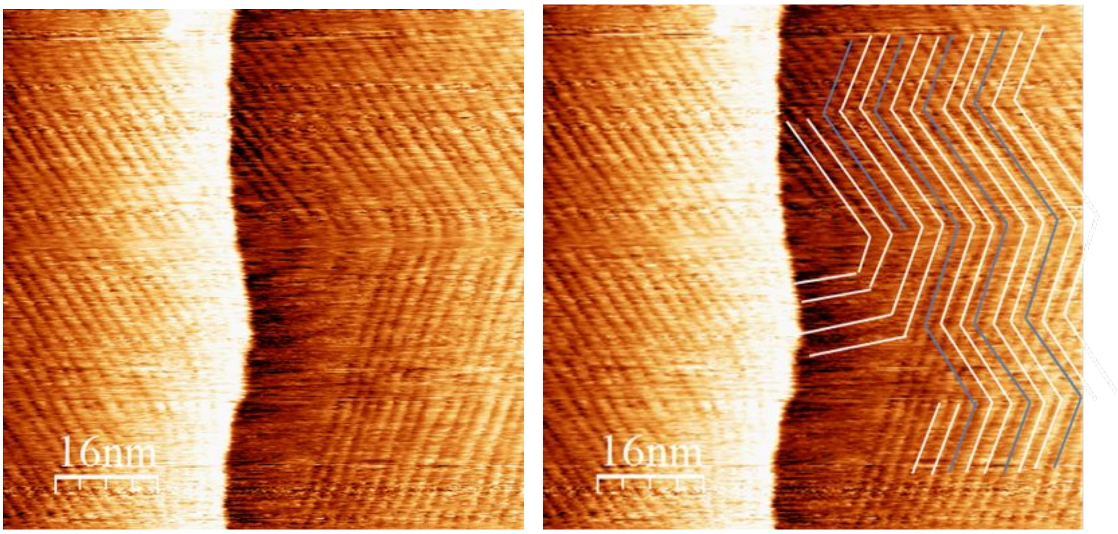

Figure S1. STM image after evaporation of 25s (coverage 0.3ML). The **BDAI** molecules arrange in rows following the gold reconstruction lines, therefore at the $[11\bar{2}]$ directions. The gold reconstruction can be observed in the center of the image at the lower terrace and underneath the molecular rows at the top of the image. Some of the reconstruction lines are depicted at the right image: in white the reconstruction lines and in blue the intermedia (fcc) areas. Notice at the right lower side that the molecular rows extend longer that the reconstruction lines. Size (80.0nm x 80.0nm).

At low coverage the **BDAI** molecules grow in rows preferentially following the Au(111) herringbone lines. Three molecular rows fit inside the gold reconstruction cell (Figure S2), producing extra spots in the LEED diagram outside the reconstruction spots (Figure S3). Additionally, the most probable alternation of **BDAI** enantiomers induces a six-row cell (rows in orange in the model), which produces extra spots inside the reconstruction ones. The brick-wall packing areas, with homochiral molecules, contribute to the external spots.

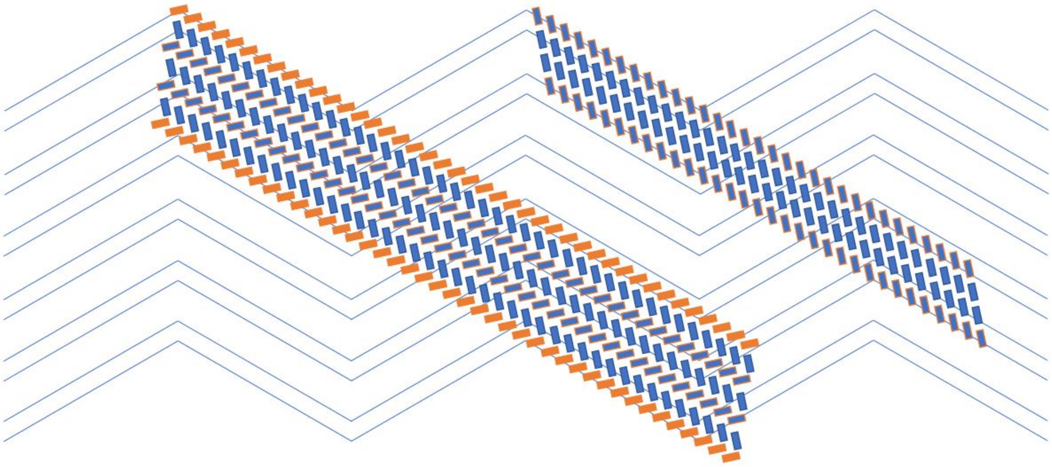

Figure S2. Model of the organic rows inducing new periodicities. The rows preferentially follow the reconstruction lines (zig-zag lines in blue). Two molecular rows over the reconstruction lines and a third row between these lines at the wider area (fcc). This registry produces a six-rows periodicity (orange rows). At the right side, the homochiral area produces a short periodicity.

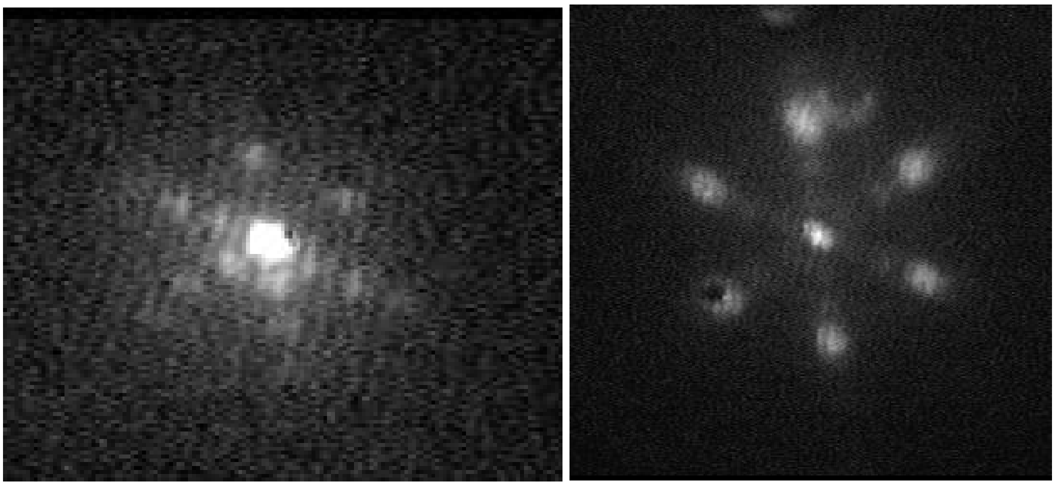

Figure S3. LEED diagrams at different coverages showing the extra spots observed close to the reconstruction ones.

The brick-wall packing areas only contribute to the external spots. Increasing the coverage results into a higher interaction between the molecular rows with a concomitant weakening of the interaction with the substrate. The assembly is then dominated by the interaction between rows.

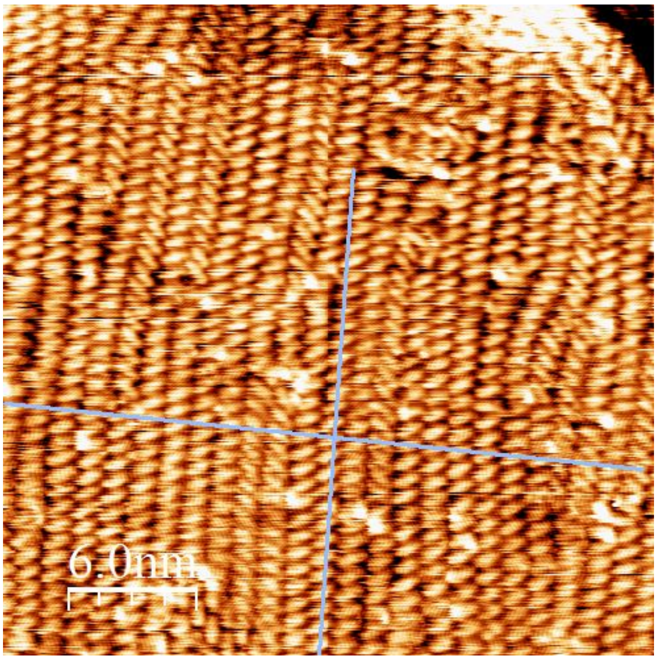

Figure S4. STM image with a higher coverage (0.7ML). The **BDAI** molecules arrange also in rows but no signature of the gold reconstruction can be seen. Size (30.0nm x 30.0nm).

Further evaporation results in a loss of the substrate registry, and the LEED spots induced by the **BDAI** molecules vanish.

XPS fitting procedure

Before the HR-XPS data were analyzed, the contribution of the backgrounds was removed by a Shirley routine. The fitting of the core levels was performed using Gaussian-Lorentzian convoluted curves. The full width half maximum (FWHM) of the Lorentzian component was fixed to 0.2 eV. While the FWHM for the Gaussian component varies from 0.5 to 0.8 eV.

Table S1. HR-XPS characterization of BTAI layers**.**

Sample	*Core level*	*Assignment*	*BE (eV)* [a]	*FWHM G/L (eV)* [b]	*%* [c]
2.0 Å	N 1s	N-pyridine	398.35	0.6/0.2	43
	N 1s	pyrrolic NH	399.13	0.7/0.2	53
	N 1s	H-pyridine*	399.85	0.6/0.2	4
0.8. Å	N 1s	N-pyridine	398.27	0.7/0.2	44
	N 1s	pyrrolic NH	399.07	0.7/0.2	49
	N 1s	H-pyridine*	399.80	0.6/0.2	7
11.7 Å at 200 ºC	N 1s	N-pyridine	398.35	0.6/0.2	40
	N 1s	pyrrolic NH	399.13	0.8/0.2	52
	N 1s	H-pyridine*	400.07	0.7/0.2	8
2.0 Å	C 1s	C-C / C-H	284.06	0.6/0.2	52
	C 1s	C-N	284.74	0.6/0.2	35
	C 1s	N-C-N	285.70	0.5/0.2	13
0.8. Å	C 1s	C-C / C-H	283.98	0.6/0.2	51
	C 1s	C-N	284.65	0.7/0.2	36
	C 1s	N-C-N	285.64	0.6/0.2	13
11.7 Å at 200 ºC	C 1s	C-C / C-H	284.08	0.6/0.2	49
	C 1s	C-N	284.77	0.6/0.2	35
	C 1s	N-C-N	285.69	0.7/0.2	16

[a] Binding energy.

[b] FWHM of the Gaussian and Lorentzian components.

[c] Percentage of the core level total area.

* Ascribed to (-NH) that does not form H bonds or hydrogenated pyridine.

NEXAFS spectra of the multilayer

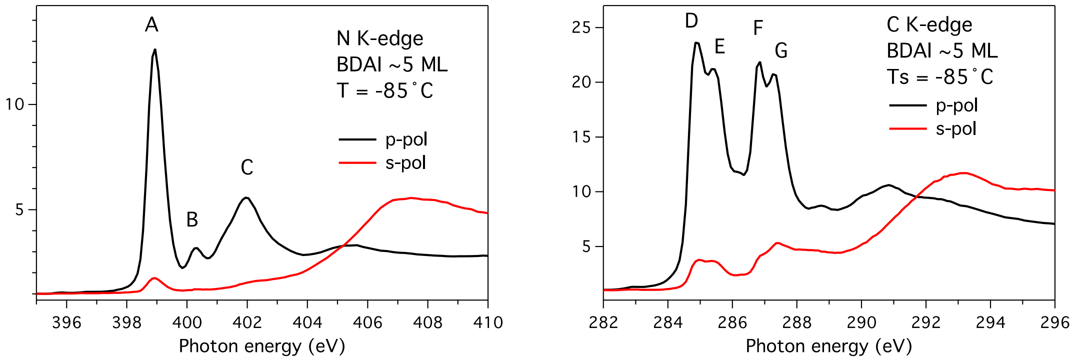

Figure S5. NEXAFS spectra of 4.7 ML (multilayer) BDAI deposited on Au(111), collected at the N K-edge and C K-edge, with the electric field of the incident photons perpendicular (p-polarization) to the surface (black line) and with the electric field of the incident photons parallel (s-polarization) to the surface (red line).

Theoretical methods

Computational details. On the basis of the experimental LEED and STM evidences, the structure, electronic properties and theoretical STM-imaging of the two different **BDAI** molecular phases on Au(111) observed have been theoretically investigated by density functional theory (DFT). We have adopted an adequate combination of the plane-wave and localized basis set DFT-based atomistic simulation packages QUANTUM ESPRESSO [S1] and FIREBALL [S2], respectively. In a first step we fully DFT-relaxed the structural configuration of both interfacial phases by using the QUANTUM ESPRESSO plane-wave code [S1]. In order to include the effect of dispersive forces into conventional DFT functionals the calculations account for an efficient semi-empirical van der Waals R^{-6} correction (DFT+D3 method [S3]). Electronic exchange and correlation effects were considered by the generalized gradient approximation GGA-PBE functional [S4]. Ultrasoft RRKJ pseudopotentials were used to model the ion-electron interaction within the H, C, N and Au atoms [S5]. The Brillouin zones were sampled using optimal Monkhorst-Pack grids [S6], and the one-electron wave-functions were expanded in a basis of plane waves with 500 eV cutoff for the kinetic energy. Atomic relaxations were performed until the maximum force acting on any atom was below 0.02 eV/Å. The Au(111) metal substrate was modeled as an infinitely periodic metal slab with four physical layers, keeping fixed the two bottommost ones during the

structural relaxation process. For the construction of the two different molecular interfacial adlayers, **BDAI** molecules were included on the metal surface according to the experimental LEED and STM results in their optimized gas-phase geometry as an excellent starting point previous to the fully relaxation. Simulations of the STM images were performed within the Kledish-Green function formalism [S7] as implemented in the localized-basis set code FIREBALL [S2] where both sample (different interfaces) and scanning tip (W-tip in this case) contributions are explicitly considered.

Core level shift calculations. Calculations of N 1s core level binding energy shifts (CLS) have been performed with the plane-wave code QUANTUM ESPRESSO [S1] within the final state approximation [S8]. With the use of pseudopotentials (just the present case), only binding energy differences (shifts) for core states on a selected atom (in our case N 1s) are relevant for a comparison with experimental XPS observations. Within the final state approximation, the energy relaxation due to the screening of the core-hole created during the photoemission process is explicitly included. For the calculation of the final state CLS, in a first step, an all-electron pseudopotential that includes a core-hole in the level of interest (in our case the N 1s) is considered. The energy of the system is then computed including a new chemical species accounting for the N atom with the core-hole. In the case of molecules with no free-electrons reservoir (unlike metal bulks or surfaces) the extra electron removed from the pseudopotential is compensated by adding an extra positive charge to the electronic background of the system. The corresponding final state CLS is then obtained as the difference between the system energy with (E_{hole}) and without (E_{ground}) the core hole, measured with respect to a reference system:

$$CLS_{final} = [E_{hole} - E_{ground}] - [E_{hole} - E_{ground}]^{ref}. \qquad \text{(S1)}$$

For a given atom of interest, one just needs to compute the total energy difference of the system with a core hole in that chosen atom, with respect to the total energy of the system with a core hole in the atom of reference. A dense grid of k-points to obtain converged results up to 0.01 eV in total energy differences has been used. First-principles calculations within this approach have been successfully applied in many different studies ranging from core level shifts in small molecules, nanowires and clusters [S9, S10] to surface core level shifts [S11,S12]. Here, we investigate the N 1s CLSs induced by the intermolecular H-bond formation in both **BDAI**/Au interfacial models, and for the two inequivalent N atoms ($N_{pyrrole}$ and $N_{pyridine}$) per molecule according to their different chemical environment (Figure 1).

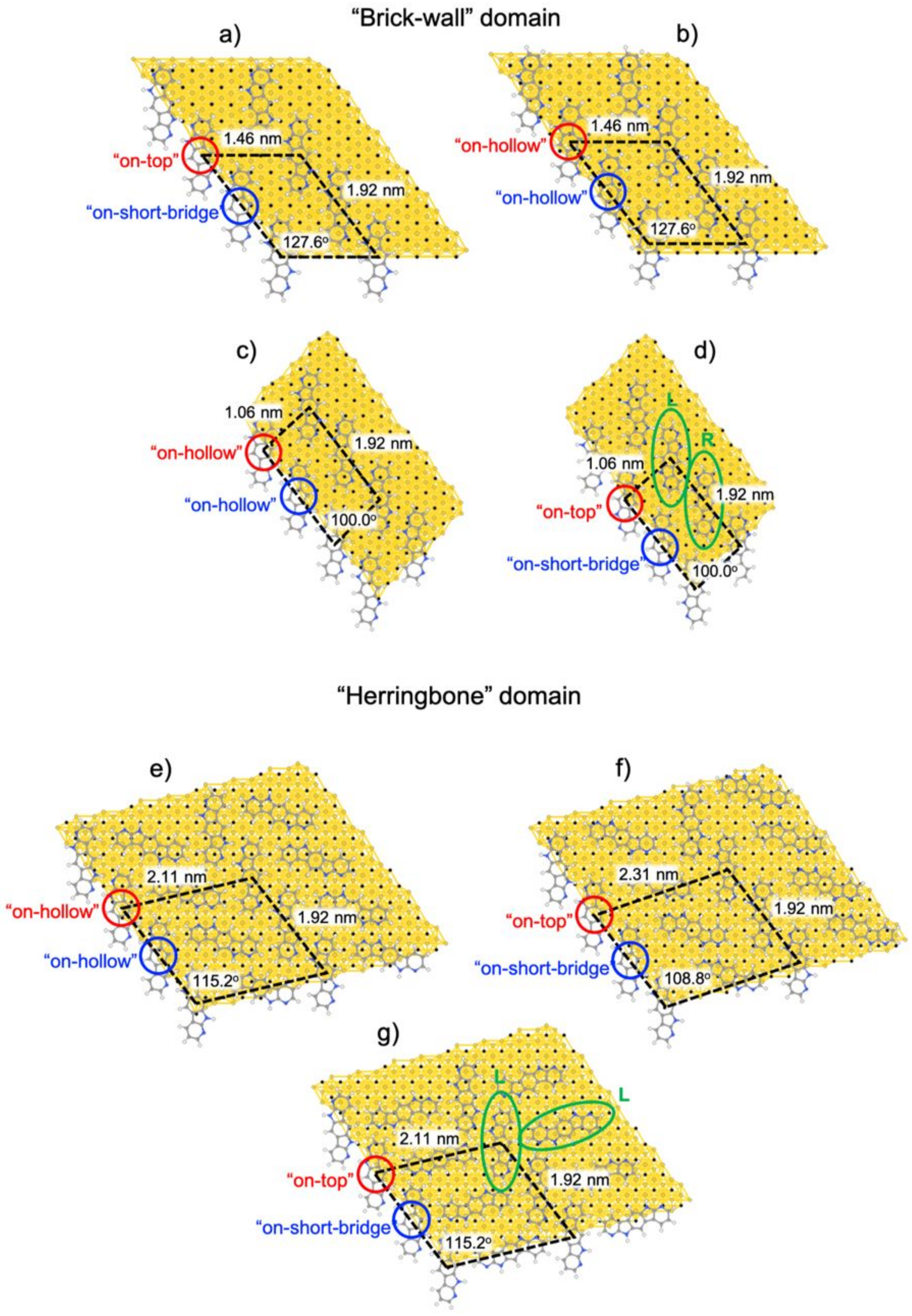

Figure S6. In the picture we show top pictorial views of some representative from more than 20 interfacial structures theoretically analyzed before the definitive candidate structures were obtained for both the "brick-wall" (a—d) and "herringbone" (e—g) domains. Structural variations w.r.t. the definitive preferential models proposed in the main text involve different on-surface adlayer adsorption site (b, c and e), different adlayer arrangement lattice (a and f), and different molecular quirality (d and g).

References

[S1] Giannozzi, P.; Baroni, S.; Bonini, N.; Calandra, M.; Car, R.; Cavazzoni, C.; Ceresoli, D.; Chiarotti, G. L.; Cococcioni, M.; Dabo, I.; Dal Corso, A.; de Gironcoli, S.; Fabris, S.; Fratesi, G.; Gebauer, R.; Gerstmann, U.; Gougoussis, C.; Kokalj, A.; Lazzeri, M.; Martin-Samos, L.; Marzari, N.; Mauri, F.; Mazzarello, R.; Paolini, S.; Pasquarello, A.; Paulatto, A.; Sbraccia, C.; Scandolo, S.; Sclauzero, G.; Seitsonen, A. P.; Smogunov, A.; Umari, P.; Wentzcovitch, R. M. Quantum Espresso: A Modular and Open-Source Software Project for Quantum Simulations of Materials. *J. Phys. Condens. Matter.* **2009,** *21,* 395502. DOI: 10.1088/0953-8984/21/39/395502

[S2] Lewis, J. P.; Jelinek, P.; Ortega, J.; Demkov, A. A.; Trabada, D. G.; Haycock, B.; Wang, H.; Adams, G.; Tomfohr, J. K.; Abad, E.; Wang, H.; Drabold, D. A. Advances and Applications in the Fireball Ab Initio Tight-Binding Molecular-Dynamics Formalism. *Phys. Status Solidi B* **2011,** *248,* 1989-2007. DOI: 10.1002/pssb.201147259

[S3] Grimme, S.; Antony, J.; Ehrlich, S.; Krieg, H. A consistent and accurate *ab initio* parametrization of density functional dispersion correction (DFT-D) for the 94 elements H-Pu. *J. Chem. Phys.* **2010,** *132,* 154104. DOI: 10.1063/1.3382344

[S4] Perdew, J. P.; Burke, K.; Ernzerhof, M. Generalized Gradient Approximation Made Simple. *Phys. Rev. Lett.* **1996,** *77,* 3865. DOI: 10.1103/PhysRevLett.77.3865

[S5] Rappe, A. M.; Rabe, K. M.; Kaxiras, E.; Joannopoulos, J. D. Optimized pseudopotentials. *Phys. Rev. B* **1990,** *41,* 1227. DOI: 10.1103/PhysRevB.41.1227

[S6] Pack, J. D.; Monkhorst, H. J. "Special Points for Brillouin-Zone Integrations" a Reply. *Phys. Rev. B* **1977,** *16,* 1748. DOI: 10.1103/PhysRevB.16.1748

[S7] Blanco, J. M.; González, C.; Jelínek, P.; Ortega, J.; Flores, F.; Pérez, R. First-principles simulations of STM images: From tunneling to the contact regime. *Phys. Rev B* **2004,** *70,* 085405. DOI: 10.1103/PhysRevB.70.085405

[S8] Pehlke, E.; Scheffler, M. Site-Sensitive Screening of Core Holes at the Si and Ge (001) Surface. *Phys. Rev. Lett.* **1993,** *71,* 2338. DOI: 10.1103/PhysRevLett.71.2338

[S9] Richter, B.; Kuhlenbeck, H.; Freund, H. -J.; Bagus, P. S. Cluster core-level binding-energy shifts: the role of lattice strain. *Phys. Rev. Lett.* **2004,** *93,* 026805. DOI: 10.1103/PhysRevLett.93.026805

[S10] García-Gil, S.; Arnau, A.; García-Lekue, A. Exploring large O 1s and N 1s core level shifts due to intermolecular hydrogen bond formation in organic molecules. *Surf. Sci.* **2013,** *613,* 102. DOI: 10.1016/j.susc.2013.03.017

[S11] Catellani, A.; Calzolari, A. Functionalization of SiC(110) Surfaces via Porphyrin Adsorption: Ab Initio Results. *J. Phys. Chem. C* **2012,** *116,* 886. DOI: 10.1021/jp209072n

[S12] Bueno, R. A.; Martínez, J. I.; Luccas, R. F.; Ruiz del Árbol, N.; Munuera, C.; Palacio, I.; Palomares, F. J.; Lauwaet, K.; Thakur, S.; Baranowski, J. M.; Strupinski, W.; López, M. F.; Mompean, F.; García-Hernández, M.; Martín-Gago, J. A. Highly selective covalent organic functionalization of epitaxial graphene. *Nature Commun.* **2017,** *8,* 15306. DOI: 10.1038/ncomms15306
